# Cartilage Intermediate Layer Protein‐1 Promotes Extracellular Matrix Degeneration via Interacting With CD47

**DOI:** 10.1111/jcmm.70506

**Published:** 2025-03-23

**Authors:** Jiezhong Deng, Yusheng Yang, Yu Xiang, Fei Luo, Jianzhong Xu, Zehua Zhang, Jinyue He

**Affiliations:** ^1^ Department of Orthopedics Southwest Hospital, Army Medical University Chongqing China

**Keywords:** cartilage intermediate layer protein‐1 (CILP‐1), CD47, intervertebral disc degeneration (IDD), P38

## Abstract

Intervertebral Disc Degeneration (IDD) is a multifactorial result contributing to Low Back Pain (LBP) while Cartilage Intermediate Layer Protein‐1 (CILP‐1) is gradually up‐regulated along with IDD. Whether CILP‐1 acts in a direct role promoting IDD via regulating matrix metabolism remains to be elucidated. Herein, we firstly detected the expression level of matrix‐related phenotypes in nucleus pulposus (NP) cells treated with CILP‐1, including ADAMTS, MMPs, IL‐6, Collagens, Aggrecan (ACAN) and SOX9. Meanwhile, the phosphorylation levels of MAPKs and NF‐κB were detected to explore the involved signalling pathways, which were further validated by inhibition experiments. Furthermore, molecular docking analysis was employed to evaluate the possibility of CD47 acting as the direct receptor mediating CILP's regulation above, which was further validated by immunoprecipitation and inhibition experiment. Our findings have made a comprehensive investigation into the regulatory effect of CILP‐1 on the matrix metabolism of NP cells and explored the underlying mechanism.

## 1 Introduction

Intervertebral disc degeneration (IDD) has been recognised as the main pathology contributing to Low Back Pain (LBP) which has resulted in an enormous socio‐economic burden worldwide. Multiple factors have been reported to be involved in the aetiology, including excessive mechanical loading, nutritional deficiency, aging, smoking, trauma et al. [1, 2, 3, 4]. Though the complicated mechanisms underlying the aetiology remain to be illustrated, there is a growing consensus that severe IDD is generally characterised by decreased disc height and torn annulus fibrosus, accompanied by impaired segmental stability, with an enhanced risk of radiculopathy, in which the disruption of extracellular matrix (ECM) homeostasis plays a pivotal role in promoting the whole IDD. ECM is the composite of multiple matrix components synthesised and secreted by NP cells residing in NP tissues, constituting the microenvironment critical in maintaining the normal metabolism of NP cells, as it allows necessary nutritional and signal interactions. ECM homeostasis is maintained by balanced matrix metabolism between anabolism and catabolism [5]. Once degeneration is initiated, catabolic proteases including MMPs and ADAMTS have been identified as significantly up‐regulated and accumulated alongside degeneration, along with an enhanced level of pro‐inflammatory cytokines such as ILs and TNF‐a [6]. Meanwhile, normal matrix components including ACAN and COL2A1 have been found suppressed in expression, leading to a significant discrepancy between matrix anabolism and catabolism. As the imbalanced matrix metabolism progresses to an irreversible degree, general physical conditions will be profoundly altered, including decreased pH values, increased osmotic pressure, and hypoxia, which are detrimental to the normal metabolism of NP cells [7, 8], forming a vicious cycle. Therefore, it is of great importance to identify the critical factor mediating the matrix degradation and to make effective interference to restore the normal matrix homeostasis.

Cartilage Intervertebral Layer Protein‐1 (CILP‐1) is an extracellular protein composed of 1184 amino acids synthesised by the NP cells with a molecular mass of 132.5KDa, mainly present in deep NP tissues [9]. The precursor of CILP‐1 is complex, including a putative signal peptide and two distinct polypeptides. Classical CILP‐1 mainly refers to the N‐terminus. Growing evidence has revealed the presence and possible role of CILP‐1 in degenerative diseases in articular cartilage, myocardium and discs [10, 11]. Meanwhile, CILP‐1 has been detected up‐regulated significantly along with osteoarthritis and revealed to be an important contributor to the initialisation and development of osteoarthritis [12], while the underlying mechanism remains to be illustrated. Given the significantly up‐regulated expression in IDD, it is reasonable to suspect that CILP‐1 may play an important role in IDD's pathology due to the reasons below: (i) Expression specificity: CILP‐1 is exclusively expressed in limited tissues, including intervertebral discs, joints cartilage, and heart muscles, and up‐regulated significantly with degeneration in the tissues above [10, 11]; (ii) Degeneration correlation: a single nucleotide polymorphism in the CILP‐1 gene sequence has been recognised as correlated with IDD in some human races [13]; Meanwhile, obvious degeneration signs have been detected by MRI in transgenic mice overexpressing CILP‐1 [14]. The evidence above suggests CILP‐1 may be an important contributor in IDD. Nevertheless, there is a paucity of comprehensive investigation into the detailed effect of up‐regulated CILP‐1 on the matrix regulation of NP cells.

In the present study, we firstly comprehensively examined the direct regulatory effect by CILP‐1 on the matrix metabolism of NP cells, including pro‐anabolic and pro‐catabolic cytokines and proteases, to validate whether up‐regulated CILP‐1 acts as a direct contributor to IDD by promoting matrix degradation. Secondly, we examined whether the regulation by CILP‐1 above is mediated by binding to CD47, a membrane receptor suggested by our molecular docking analysis. Lastly, we investigated into the downstream signalling pathway mediating CILP‐1's regulation on matrix metabolism above. Through the research, we aimed to make a primary study about CILP‐1's regulatory effect on matrix metabolism of NP cells and explore the underlying mechanisms.

## 2 Methods and Materials

### 2.1 Cell Lines and Primary Cultures

Human intervertebral disc (IVD) tissues had been collected from 30 patients (M/F: 1) ranging between 20 and 45 years in age, who had undergone discectomy for IDD at our orthopaedic department from January 2022 to December 2023. Pre‐operative MRI had been employed to confirm the Pfirrmann grade was less than III. Once removed during the operation, the IVD tissue was conserved in sterile saline solution and sent to the laboratory immediately. Transferred into a sterile culture dish, the gel‐like NP tissue was meticulously isolated from the annulus fibrosus and minced into small particles less than 1 mm in diameter, which were then incubated with 0.2% type II collagenase in Dulbecco's modified Eagle's medium (DMEM)/F‐12 medium (Invitrogen Life Technologies, Carlsbad, CA, USA) at 37°C for 12 h after washing with PBS three times. The suspension obtained was collected and centrifuged at 1200 rpm for 6 min to obtain concentrated NP cells, which were subsequently transferred to DMEM/F‐12 containing 10% foetal bovine serum (FBS) and 1% penicillin/streptomycin (Invitrogen Life Technologies). Placed in a 37°C incubator with 5% CO_2_, the original NP cells were cultured in the medium above and refreshed every 3 days during the period. Once reaching 80% confluence, the original passage of NP cells was sub‐cultured into more dishes. Notably, original NP cells from different patients were pooled together at passage 1 and co‐cultured at passages 2 and 3 prior to subsequent experiments. Pooled NP cells would be validated by using qPCR with three positive marker genes [15] including CA12, KRT19, and CDH2, and a negative marker (IBSP) before any treatments.

### 2.2 Method Details

#### 2.2.1 Ethnic Statement

The present study was performed in compliance with the Declaration of Helsinki and approved by the Ethics Committee of our institution.

#### 2.2.2 Cell Counting Kit‐8 Test

Cells were distributed into a 96‐well plate and treated with 0, 10, 100, and 1000 ng/mL CILP‐1 for 96 h. Then, 10 μL CCK‐8 reagent was added to each well, and cells were continued to incubate for 2 h. The absorbance at a wavelength of 450 nm was measured with an enzyme label.

#### 2.2.3 Treatment With High‐Concentration CILP‐1

To examine the comprehensive effect of CILP‐1 on matrix homeostasis of NP cells, sub‐confluent NP cells were equally divided into a treatment group and a control group at a density of 5 × 10^6^well. The treatment group was treated with CILP‐1 (1000 ng/mL). Cells were incubated at 37°C and 5% CO_2_ for 96 h.

#### 2.2.4 Real‐Time PCR

The RNA‐Quick Purification kit (Yishan, Shanghai, China) was employed to isolate the total RNA from NP cells. A total of 1 μg RNA was used for reverse transcription by using the ABScript Neo RT Master Mix for qPCR with gDNA Remover (ABclonal, Wuhan, China) according to the protocol. DNA was amplified by qPCR using the 2X Universal SYBR Green Fast qPCR Mix (ABclonal, Wuhan, China) and a Real‐Time PCR system (Biorad, California, USA). A 10 μL reaction volume was used as below: initial heat activation for 30 s at 95°C, followed by 40 cycles of 5 s at 95°C for template denaturation and 30 s at 60°C for annealing and extension. All samples were amplified in triplicate, with results presented as Ct values. The expression level of the targeted genes was normalised to that of GAPDH. Relative mRNA expression levels of target genes were calculated using the 2^−ΔΔCt^ method. The sequences of the primers were displayed in Table S1.

#### 2.2.5 Western Blotting

Total proteins were extracted by RIPA (Beyotime, Shanghai, China) with Halt Protease/Phosphatase Inhibitor (Thermo Fisher Scientific, Massachusetts, USA) at 4°C, with frequent agitation for 30 min. Cell lysates were removed of insoluble debris by centrifugation at 12,000 *g* for 15 min at 4°C. The amount of total protein was determined by the BCA Protein Assay Kit (Beyotime, Shanghai, China). Equal amounts of protein (30 μg) were separated by 10% SDS‐PAGE and transferred onto polyvinylidene difluoride membranes (EMD Millipore, Massachusetts, USA), which were blocked with 5% BSA for 2 h at room temperature. The filters were incubated overnight at 4°C with primary antibodies diluted 1:1000 and then with horseradish peroxidase conjugated secondary antibodies (CST, Massachusetts, USA) diluted 1:2000 for 1.5 h at room temperature. Bands were detected and scanned by using a Gel Imager (Biorad, California, USA).

#### 2.2.6 Immunoprecipitation

Whole cell lysates of NP cells were extracted as the negative control group. Immunoprecipitation of NP cells treated with or without CILP‐1 was conducted according to the instructions of the rProtein A/G Magnetic IP/Co‐IP Kit (ACE Biotechnology, Nanjing, China) by using IgG and anti‐CD47 antibodies. Western blot was performed to detect the expression of CILP‐1 and CD47.

#### 2.2.7 Treatment With CD47 Inhibitor

To validate whether the direct interaction above has mediated CILP‐1's regulation of matrix metabolism, NP cells in the treatment group were pre‐treated with the CD47 inhibitor RRx‐001 (5 μM) for 24 h, then incubated with CILP‐1 for 96 h prior to the total RNA being extracted for qPCR and total protein for western blot. NP cells treated with CILP‐1 exclusively served as the control group.

#### 2.2.8 Downstream Signalling Pathway Exploration

To explore the downstream signalling pathway mediating the matrix regulation after CD47 activation, the phosphorylation of p38 and NF‐κB was detected by western blot after NP cells were treated with CILP‐1 for 96 h. To further confirm the role of the activated pathway in mediating CILP's regulation on matrix metabolism, NP cells were pre‐treated with a p38‐specific inhibitor PD 169316 for 24 h prior to incubation with CILP‐1 for 96 h, then total RNA and protein were extracted for qPCR and western blot.

#### 2.2.9 Cellular Immunofluorescence Staining

Chondrocytes and NP cells were seeded into a 48‐well plate and incubated for 24 h. After fixation, cells were incubated with anti‐KRT19 antibody overnight at 4°C. After that, cells were incubated with AF647‐secondary antibody and DAPI. Images were captured by the fluorescence microscope.

#### 2.2.10 Tissue Paraffin Section Staining

The nucleus pulposus tissues were sliced into 5 μm thick sections. Haematoxylin and eosin (H&E) staining, and immunofluorescence/immunohistochemistry staining for CILP‐1 and CD47 were then performed according to the instructions, and sections were scanned by Pannoramic MIDI.

#### 2.2.11 Quantification and Statistical Analysis

All experiments had been repeated at least three times. The data was presented as the mean ± standard error. The RT‐qPCR data was analysed by *t* tests. GraphPad Prism 6 was used to analyse the data obtained. *p* < 0.05 was regarded as statistical significance.

## 3 Results

### 3.1 NP Cell Validation

**FIGURE 1:**
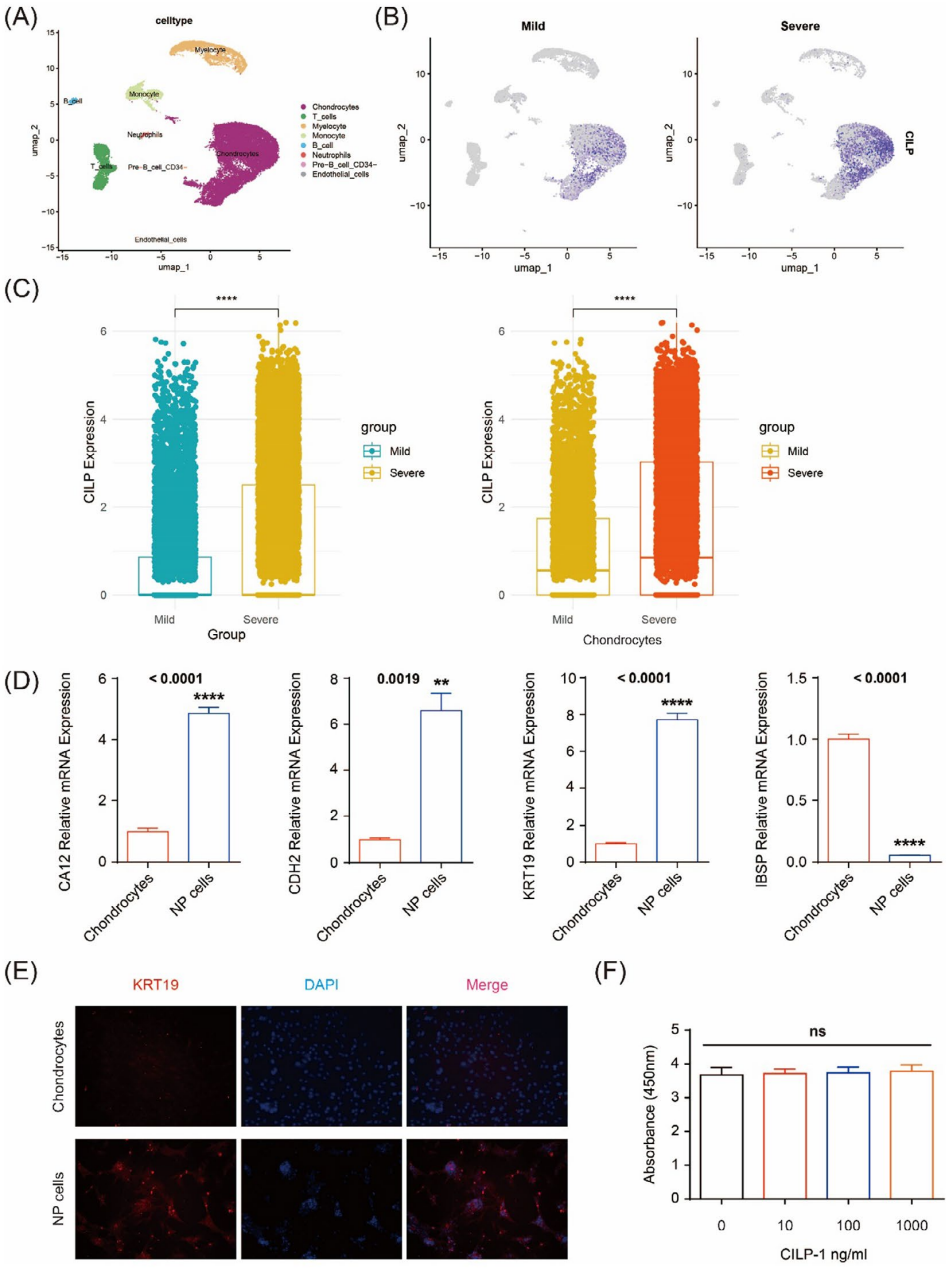
Validation of NP cells and cell viability. (A) The UMAP plot of single‐cell sequencing clustering results; (B) the expression of CILP through the UMAP plot; (C) the expression of CILP through the boxplot; (D) validation of NP cells by PCR; (E) validation of NP cells by IF; (F) the toxicity test of CILP (0, 10, 100, 1000 ng/mL) on NP cells.

Firstly, we analysed the datasets consisting of 4 mildly (Pfirrmann II–III) and 4 severely (Pfirrmann IV‐V) degenerative human IVDs (GSE165722). The meta‐analysis identified 11 cell clusters from a total of 22,453 cells, and the cell types were annotated based on their cluster differentially expressed genes (Figures 1A; S4A–D). We displayed the expression of CILP through the UMAP plot, and the results showed that the expression in the severe group was higher than that in the mild group (Figure 1B). The boxplot results intuitively demonstrated that the expression of CILP was significantly higher in the severe group than in the mild group, and this was predominantly observed in chondrocytes (Figures 1C; S4E). NP cells collected have been confirmed positive in CA12, CDH2, and KRT19 gene expression and negative in IBSP gene expression by qPCR, complying with typical NP cell characteristics (Figure 1D,E) [15]. Cell viability has been determined by CCK8, and it has been shown that 1000 ng/mL CILP‐1 had no effect on the proliferation of NP cells (Figure 1F).

### 3.2 CILP‐1 Up‐Regulated Pro‐Catabolic Phenotypes and Down‐Regulated Pro‐Anabolic Concurrently

**FIGURE 2:**
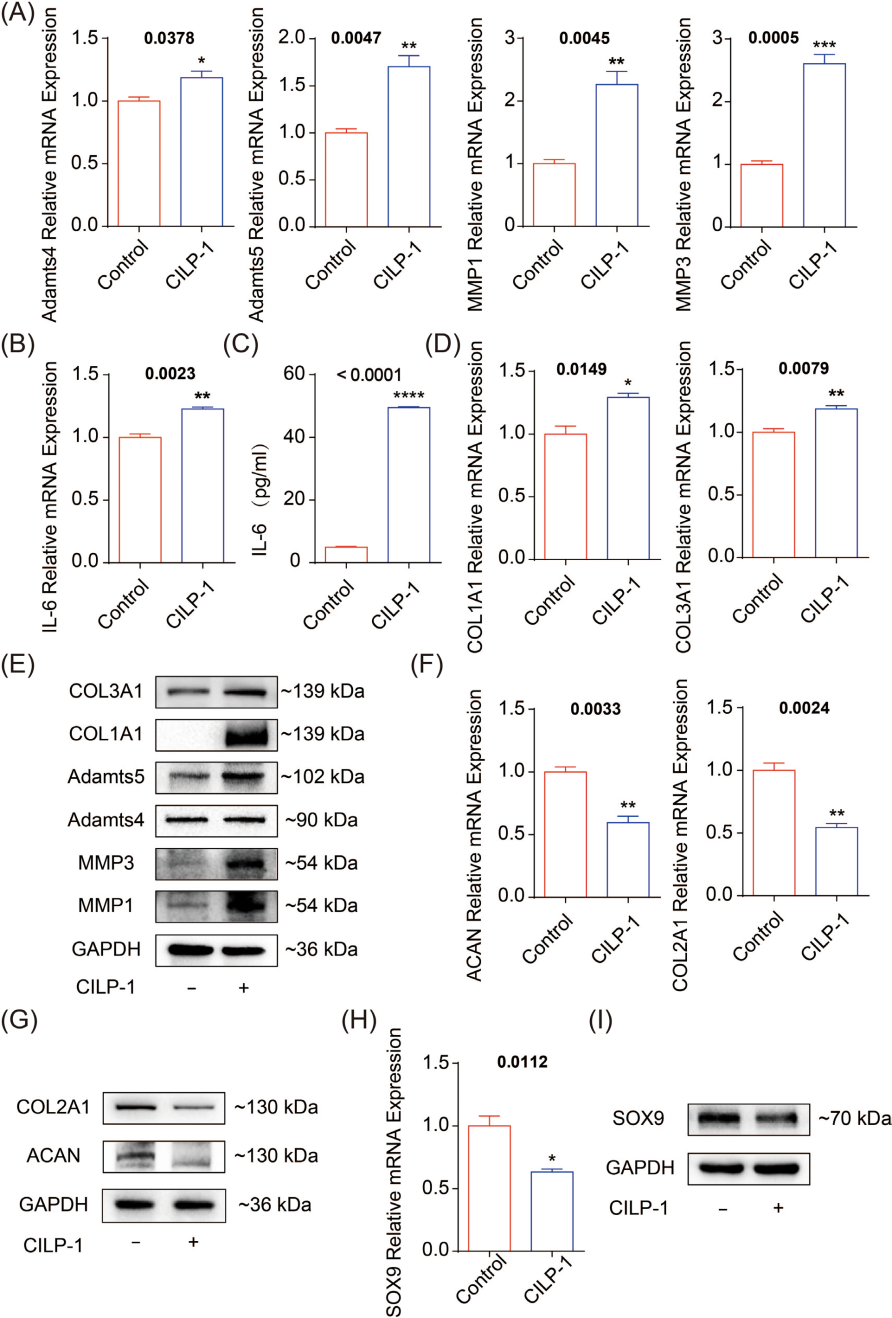
Matrix regulation of NP cells by CILP‐1. (A) Gene expression of ADAM‐4/5 and MMP‐1/3; (B) gene expression of IL‐6; (C) protein expression detected by ELISA; (D) gene expression of COL1A1/3A1; (E) protein expression of Col1A1/3A1, ADAM‐4/5 and MMP‐1/3 detected by western blotting; (F, G) gene and protein expression of ACAN and Col2A1; (H, I) gene and protein expression of SOX9.

We firstly investigated the regulatory effect of CILP‐1 on matrix homeostasis. NP cells treated with CILP‐1 for 72 h were collected as the experimental group, while NP cells without any treatment were used as the control group. Consequently, we observed that the cytoskeleton of NP cells changed after treatment with CILP‐1 (Figure S1). The qPCR results showed that the mRNA expression of pro‐catabolic proteases including ADAMTS4/5 and MMP1/3 was significantly increased after the treatment of CILP‐1 (Figure 2A). Additionally, the pro‐inflammatory cytokine IL‐6 was obviously up‐regulated in mRNA expression (Figure 2B). Meanwhile, ELISA results also demonstrated significantly increased secretion of IL‐6 (Figure 2C). Moreover, the fibrosis phenotypes including COL1A1 and COL3A1, which should be more common in the annulus fibrosus, were significantly up‐regulated in mRNA expression (Figure 2D). Accordingly, the protein expression of ADAMTS4/5 and MMP1/3 was significantly increased, along with enhanced fibrosis phenotypes including COL1A1 and COL3A1(Figure 2E), complying with the gene regulations above.

With respect to the pro‐anabolic phenotypes, the results demonstrated the normal matrix phenotypes ACAN and COL2A1 were significantly suppressed in mRNA and protein expression after CILP‐1 treatment (Figure 2F,G). Meanwhile, the pro‐anabolic transcription factor SOX9 was also greatly decreased in mRNA and protein expression (Figure 2H,I).

### 3.3 Membrane Receptor CD47 Mediated the Negative Regulation of Matrix Metabolism by CILP‐1

**FIGURE 3:**
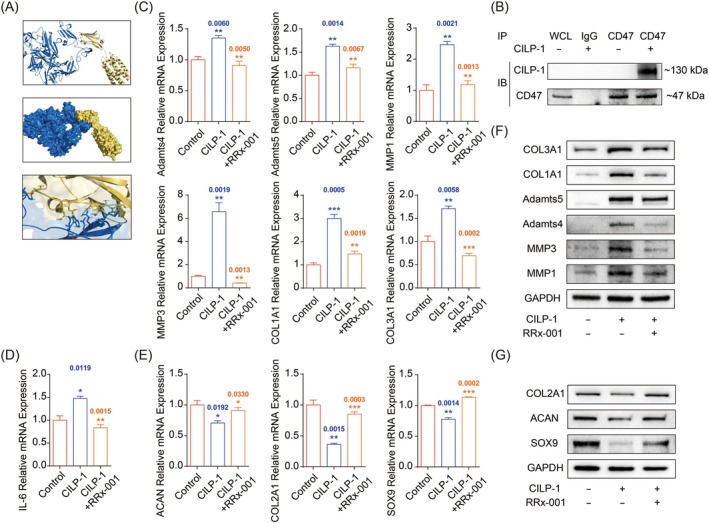
CD47 mediates matrix regulation by CILP‐1. (A) Molecular docking analysis suggesting a possible direct binding effect; (B) Co‐immunoprecipitation validated the direct binding effect; (C) Gene expression of pro‐catabolic phenotypes with/without CD47 inhibited; (D) Gene expression of IL‐6 with/without CD47 inhibited; (E) Gene expression of pro‐anabolic phenotypes with/without CD47 inhibited; (F, G) Protein expression of the phenotypes above.

CD47 was a common membrane receptor mediating multiple signalling transductions in NP cells and a possible direct binding receptor suggested by our molecular docking analysis (Figure 3A). The staining results of tissue slices showed that CILP‐1 and CD47 were upregulated and co‐localised in degenerative intervertebral disc tissue, further indicating the interaction between CILP‐1 and CD47 (Figure S2). To validate the hypothesis, co‐immunoprecipitation was employed, and the result showed CD47 was capable of directly binding to CILP‐1, suggesting the membrane receptor might be involved in the CILP‐1‐mediated matrix regulation (Figure 3B). To further validate the hypothesis, NP cells were pre‐treated with RRx‐001, a special CD47 inhibitor, prior to CILP‐1 treatment. Consequently, qPCR results showed the up‐regulated pro‐catabolic proteases (ADAMT4/5 and MMP1/3) and abnormal fibrosis phenotypes (COL1A1 and 3A1) induced by CILP‐1 were significantly decreased in mRNA expression (Figure 3C). Similarly, the up‐regulation in IL‐6 gene expression by CILP‐1 treatment was also distinctively retarded (Figure 3D). Meanwhile, the suppressed expression of normal matrix phenotypes (ACAN and COL2A1) and pro‐anabolic transcription factor SOX9 by CILP‐1 were significantly reversed simultaneously (Figure 3E). Accordingly, western blot results showed the protein expression of pro‐catabolic proteases and fibrosis phenotypes including ADAMT4/5 and MMP1/3 and COL1A1 and COL3A1, up‐regulated by CILP‐1 treatment, were significantly suppressed by the pre‐treatment of RRx‐001 (Figure 3F). Meanwhile, the inhibition of ACAN, COL2A1, and SOX9 by CILP‐1 was also significantly reversed in protein expression by the pre‐treatment of the CD47 inhibitor (Figure 3G). All results above indicated CD47 mediated the negative regulation of matrix metabolism by CILP‐1 through a direct binding effect.

### 3.4 P38 Signalling Pathway Was Involved in the Matrix Regulation by CILP‐1

**FIGURE 4:**
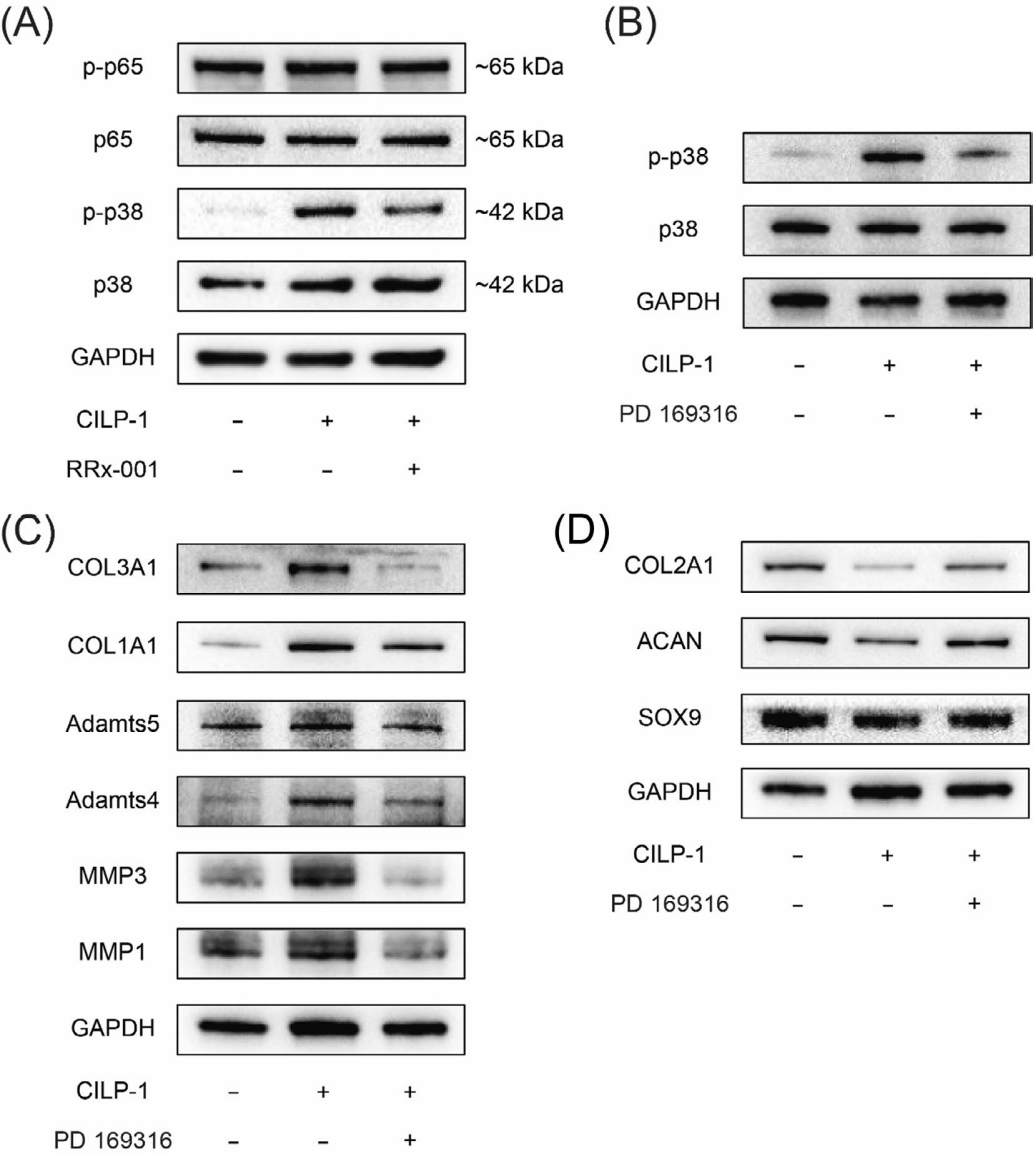
P38 signalling mediates CILP‐1′ matrix regulation. (A) The phosphorylation level of p38 in NP cells treated with CILP‐1, combined with or without CD47 inhibitor; (B) The phosphorylation level of p38 in NP cells treated with CILP‐1, combined with or without p38 inhibitor; (C, D) The protein expression of pro‐catabolic/anabolic phenotypes in NP cells treated with CILP‐1, combined with or without p38 inhibitor.

To explore the underlying pathways mediating the matrix regulation above by the CILP1‐CD47 axis, various signalling pathways were detected in NP cells treated with CILP‐1 and the CD47 inhibitor (Figure S3B). Consequently, western blot results showed that p38 was obviously phosphorylated in the experimental group (Figure 4A), indicating the possible involvement of the signalling pathway mediating CILP‐1's pro‐degeneration effect. To further validate the hypothesis, a specific p38 inhibitor PD 169316 was added to the culture medium 12 h prior to CILP‐1 treatment. As a result, not only was the phosphorylation of p38 significantly suppressed by the specific p38 inhibitor (Figure 4B), but the CILP‐1‐mediated regulatory effect on matrix‐related genes was also obviously retarded in protein expression, including ADAMT4/5, MMP1/3, SOX9, collagens, and ACAN, suggesting that the matrix regulation effect by CILP‐1/CD47 on NP cells was mainly mediated by the p38 pathway (Figure 4C).

## 4 Discussion

In the present study, we demonstrated that CILP‐1 up‐regulated pro‐catabolic proteases but inhibited pro‐anabolic transcription factors and normal matrix phenotypes concurrently via the p38 signalling pathway, through directly binding to the membrane receptor CD47. Prior studies had suggested CILP‐1 might have played an important role in the aetiology of IDD, with the detailed mechanism remaining unknown. Our results confirmed CILP‐1 could contribute to NP tissue degeneration through disrupting the balance of matrix anabolism and catabolism directly. Furthermore, we investigated the direct membrane receptor and the downstream signalling pathway mediating the regulation above. This is a comprehensive investigation into the direct regulatory effect on ECM metabolism haemostasis by CILP‐1 and the underlying mechanism.

LBP was recognised as the common musculoskeletal disorder in the world, present in 70%–80% of adults at some time in their lifetime, resulting in heavy socioeconomical burdens. Complicated aetiologies have been involved in the initialization and development of LBP. As the largest avascular and aneural tissue in the body, lumbar discs are comprised of three distinctive structures, including endplates, annulus fibrosus (AF) and nucleus pulposus (NP), in which the NP tissues act as critical structures buffering mechanical loading and maintaining the stability of the whole spine. Normal NP tissue is abundant in hydrophilic ACAN and COL2A1, which is maintained by balanced matrix metabolism of NP cells. The haemostasis of the matrix is mainly determined by the balance between anabolism and catabolism. The former consists of normal matrix components, including ACAN and COL2A1, which is maintained by pro‐synthesis cytokines such as SOX9 [16]. The latter is mainly comprised of ADAMTS and MMPs, the main catabolic proteases in NP tissue responsible for matrix component degradation. Inflammatory factors are also important contributors to ECM disruption due to their inducible effect on cell apoptosis and senescence [17]. Increased pro‐catabolic proteases, combined with inflammatory cytokines along with suppressed matrix expression, result in progressive deterioration of matrix homeostasis, accelerating IDD. Therefore, it is critical to target the core factor mediating matrix degeneration above and make necessary interference to restore matrix homeostasis.

Cartilage intermediate layer protein‐1 is a monomeric glycoprotein expressed by NP cells residing mainly in humans' IVD and articular cartilages. Noteworthy is that CILP‐1 is among the few matrix phenotypes whose expression is up‐regulated along with degeneration, indicating its possible role in the development of IDD. Further research has confirmed that one of the single nucleotide polymorphisms was genetically associated with the IDD, indicating its genetic association with degeneration pathology. Additionally, Seki et al. detected early imaging signs indicative of degeneration in MRI in transgenic mice overexpressing CILP‐1, further suggesting the promotive role of CILP‐1 in the degeneration mechanism [14]. Therefore, it has triggered great interest to investigate the possible pro‐degeneration effect of CILP‐1 given its high expression in degenerative tissues. TGF‐β has been regarded as an important factor due to its role in the synthesis of proteoglycans and cell proliferation. Studies have demonstrated that CILP‐1 could restrict the accessibility of TGF‐β to its membrane receptors by forming a complex with TGF‐β [18]. Further analysis suggested a well‐defined consensus sequence—WSXW motif in CILP‐1 may be responsible for the interaction. Meanwhile, insulin‐like growth factor‐1 is a key factor in maintaining ECM homeostasis owing to its role in ECM biosynthesis. Studies have shown that CILP‐1 was capable of counteracting the IGF‐1‐mediated chondrocyte proliferation and proteoglycan synthesis while the detailed mechanism remained to be illustrated [19]. Nevertheless, it remains unknown whether CILP‐1 promotes IDD by negatively affecting the matrix metabolism of NP cells directly. In our study, we firstly investigated whether the matrix metabolism was negatively affected by CILP‐1 treatment. Consequently, pro‐catabolic proteases including ADAMTS4/5 were significantly up‐regulated in gene and protein expression after NP cells were treated with CILP‐1 in a high concentration. Meanwhile, the MMP1/3 were also up‐regulated in mRNA and protein expression. ADAMTS and MMPs were the main matrix catabolic proteases responsible for matrix degradation; accumulated ADAMTS and MMPs in NP tissues would accelerate the degradation of the matrix structure [20, 21]. Meanwhile, as shown in our results, ACAN and COL2A1, the main components in NP tissues, were significantly suppressed in gene and protein expression after CILP‐1 treatment, along with the pro‐anabolic transcription factor SOX9. Herein, we thought that CILP‐1 accumulated in degenerative disc was capable of promoting matrix disruption in turn by promoting pro‐catabolic proteases while suppressing pro‐anabolic phenotypes concurrently, exacerbating the imbalance between matrix anabolism and catabolism. Furthermore, we also found that fibrotic components including COL1A1 and COL3A1 were obviously up‐regulated, which should be more common in AF rather than NF [22]. Fibrosis was a typical characteristic of severe degenerative disc. The normal fibrotic component COL2A1 was gradually replaced by COL1A1 and COL3A1 along with degeneration. Consequently, the changed fibrotic constitution combined with reduced ACAN resulted in an altered physicochemical environment manifested in lower pH values, reduced osmotic pressure, and decreased resilience, further exacerbating the microenvironment for the residing cells. In addition, we also detected enhanced expression and release of IL‐6, a main inflammatory cytokine in degenerated disc [23], suggesting the inflammatory response was also involved in the CILP‐1‐mediated matrix disruption. Therefore, based on our results above, it could be concluded that accumulated CILP‐1 along with degeneration in IDD could contribute to matrix degradation directly, forming a vicious cycle, which is mediated via the following aspects: (i) up‐regulating pro‐catabolic proteases; (ii) suppressing normal matrix components; (iii) promoting abnormal collagen secretion; (iv) activating inflammatory response.

It is still unknown how CILP‐1 affected NP cells' matrix regulation as an extracellular protein. Therefore, it is necessary to spot the direct receptor mediating CILP‐1's regulation. Due to the lack of research investigating potential mediators of CILP‐1's effects on NP cells. We initially conducted an analysis of the protein structure of CILP‐1 to identify possible interaction sites. Consequently, Thrombospondin type 1 (TSP‐1), a special domain in CLIP‐1, has triggered our interest secondary to its potential capability for interacting with membrane acceptors. Reviewing the potential candidates and relevant literature, we tried to identify the most likely candidate involved in matrix regulation. Based on this review above, we selected CD36 and CD47 as the primary candidates for further validation. CD47, also named the integrin‐associated protein, is widely expressed on the cellular membrane of diverse cells with a molecular weight of approximately 47 kDa. It consists of an N‐terminal extracellular IgV domain, five transmembrane helices, and a C‐terminal cytoplasmic tail region. Due to its binding effect to thrombospondin‐1(TSP‐1), CD47 has been reported to participate in various critical transduction pathways as a direct membrane receptor [24]. Julovi SM et al. has confirmed blocking TSP‐1/CD47 signalling could greatly alleviate interstitial fibrosis [25]. Gwag T et al. validated that inhibiting CD47 with specific antibodies would effectively attenuate live inflammation and fibrosis [26]. Given the presence of TSP‐1 structure in CILP‐1 protein, it is possible that CD47 may act as a direct membrane receptor directly binding to CILP‐1 and get involved in the matrix regulation by CILP‐1. As shown in our results, the docking analysis demonstrated the CD47 was capable of directly binding to CILP‐1, indicating the possibility of direct binding effect. To further validate the hypothesis, co‐immunoprecipitation was employed, and the result showed that CD47(Figure 3B and S2C), rather than CD36 (Figure S3A), acts as the direct interacting receptor. Further inhibition experiments with the CD47 inhibitor showed pre‐inhibition of CD47 significantly retarded the up‐regulation of pro‐catabolic proteases and downregulation of pro‐anabolic phenotypes by CILP‐1 treatment. Based on the evidence above, it was reasonable to conclude that CD47 acted the direct binding receptor mediating CILP‐1's regulatory effect on NP cells' matrix metabolism.

Confirming the direct receptor mediating CILP‐1's regulation, our team made further investigations into which downstream signalling pathway was involved in CILP‐mediated matrix regulation. In our results, we have checked the activation of several pathways generally involved in degenerative diseases by detecting the phosphorylation of critical messengers, including NF‐κB and MAPK pathways [27, 28, 29]. Consequently, the phosphorylation of p38 was significantly enhanced after CILP‐1 treatment while other signalling pathways remained inactivated. p38 MAPK was one of the mitogen‐activated protein kinase (MAPK) signalling pathways associated with the pathologies of LDD. It has been well documented that activated p38 MAPK is involved in inflammatory response, cell apoptosis, and senescence by phosphorylating downstream signalling messengers. Therefore, it was reasonable to suggest the p38 MAPK pathway may act as the downstream signalling way mediating CILP‐1's matrix regulation. Further inhibition experiments with a p38 inhibitor showed the up‐regulation effect on catabolic proteases and down‐regulation effect on pro‐anabolic phenotypes by CILP‐1 were significantly retarded. Therefore, it could be concluded that the downstream p38 MAPK signalling pathway mediated CILP‐1/CD47's regulation on NP cells' matrix homeostasis.

## 5 Limitation of the Study

Though the study had given a preliminary illustration of CILP‐1's role in the development of IDD, several limitations existed deserving awareness. Firstly, despite the research having spotted the direct membrane receptor involved in CILP‐1's regulation on NP cells, it still needs further research on whether other membrane receptors exist involved in the complicated ligand‐receptor interaction; Secondly, apart from p38 signalling pathways, it remains to be elucidated whether there exist more specific signalling mechanisms mediating the regulation. Thirdly, we had used human NP cells pooled from different origins, the heterogeneous cell resources might weaken the authority of the conclusion. Lastly, we have employed rhCILP‐1 as the treatment rather than over‐expression techniques, which might be more convincing.

In conclusion, CILP‐1 could up‐regulate pro‐catabolic proteases/cytokines but down‐regulate pro‐anabolic phenotypes concurrently, accelerating the disruption of matrix metabolism. The regulation was mediated by directly binding to the membrane receptor CD47 and subsequently activating the p38 MAPK signalling pathway. Our findings contribute to a better knowledge of CILP‐1's role in the pathology of IDD, suggesting the possibility of CILP‐1 as a therapeutic target for IDD in the future.

## Author Contributions

**Jiezhong Deng:** data curation (lead), formal analysis (lead), investigation (lead), methodology (lead), writing – review and editing (supporting). **Yusheng Yang:** methodology (supporting), resources (supporting), software (supporting). **Yu Xiang:** data curation (supporting), resources (supporting). **Fei Luo:** project administration (supporting). **Jianzhong Xu:** project administration (supporting). **Zehua Zhang:** conceptualization (supporting), funding acquisition (supporting), project administration (supporting), resources (supporting), supervision (supporting), writing – review and editing (supporting). **Jinyue He:** conceptualization (lead), funding acquisition (lead), project administration (lead), resources (lead), writing – original draft (lead), writing – review and editing (lead).

## Ethics Statement

The study was approved by the ethics committee of Southwest Hospital, Army Medical University.

## Consent

Informed consent was obtained from all individual participants included in the study.

## Conflicts of Interest

The authors declare no conflicts of interest.

## Supporting information


Figure S1.

Figure S4.

Table S1.

## Data Availability

All the data and materials are available upon request.

## References

[1] D. Wang , Q. Shang , J. Mao , et al., “Phosphorylation of KRT8 (Keratin 8) by Excessive Mechanical Load‐Activated PKN (Protein Kinase N) Impairs Autophagosome Initiation and Contributes to Disc Degeneration,” Autophagy 19, no. 9 (2023): 2485–2503, 10.1080/15548627.2023.2186099, 36897022. 36897022 PMC10392755

[2] M. Serjeant , P. M. Moon , D. Quinonez , S. Penuela , F. Beier , and C. A. Séguin , “The Role of Panx3 in Age‐Associated and Injury‐Induced Intervertebral Disc Degeneration,” International Journal of Molecular Sciences 22, no. 3 (2021): 1080, 10.3390/ijms22031080, 33499145. 33499145 PMC7865929

[3] J. Tu , W. Li , P. M. Hansbro , et al., “Smoking and Tetramer Tryptase Accelerate Intervertebral Disc Degeneration by Inducing METTL14‐Mediated DIXDC1 m(6) Modification,” Molecular Therapy 31, no. 8 (2023): 2524–2542. 37340635 10.1016/j.ymthe.2023.06.010 PMC10422004

[4] J. Zhou , J. Mi , Y. Peng , H. Han , and Z. Liu , “Causal Associations of Obesity With the Intervertebral Degeneration, Low Back Pain, and Sciatica: A Two‐Sample Mendelian Randomization Study,” Frontiers in Endocrinology 12 (2021): 740200. 34956075 10.3389/fendo.2021.740200 PMC8692291

[5] I. L. Mohd Isa , S. L. Teoh , N. H. Mohd Nor , and S. A. Mokhtar , “Discogenic Low Back Pain: Anatomy, Pathophysiology and Treatments of Intervertebral Disc Degeneration,” International Journal of Molecular Sciences 24, no. 1 (2022): 208. 36613651 10.3390/ijms24010208 PMC9820240

[6] C. Zhang , S. E. Gullbrand , T. P. Schaer , et al., “Inflammatory Cytokine and Catabolic Enzyme Expression in a Goat Model of Intervertebral Disc Degeneration,” Journal of Orthopaedic Research 38, no. 11 (2020): 2521–2531. 32091156 10.1002/jor.24639 PMC7483272

[7] P. A. Vergroesen , K. S. Emanuel , M. Peeters , I. Kingma , and T. H. Smit , “Are Axial Intervertebral Disc Biomechanics Determined by Osmosis?,” Journal of Biomechanics 70 (2018): 4–9. 28579261 10.1016/j.jbiomech.2017.04.027

[8] R. He , Z. Wang , M. Cui , et al., “HIF1A Alleviates Compression‐Induced Apoptosis of Nucleus Pulposus Derived Stem Cells via Upregulating Autophagy,” Autophagy 17, no. 11 (2021): 3338–3360. 33455530 10.1080/15548627.2021.1872227 PMC8632345

[9] L. Liu , J. He , C. Liu , et al., “Cartilage Intermediate Layer Protein Affects the Progression of Intervertebral Disc Degeneration by Regulating the Extracellular Microenvironment (Review),” International Journal of Molecular Medicine 47, no. 2 (2021): 475–484. 33416131 10.3892/ijmm.2020.4832 PMC7797476

[10] Z. Wang , J. H. Kim , K. Higashino , et al., “Cartilage Intermediate Layer Protein (CILP) Regulation in Intervertebral Discs. The Effect of Age, Degeneration, and Bone Morphogenetic Protein‐2,” Spine (Phila Pa 1976) 37, no. 4 (2012): E203–E208. 21857406 10.1097/BRS.0b013e31822dcf47

[11] C. L. Zhang , Q. Zhao , H. Liang , et al., “Cartilage Intermediate Layer Protein‐1 Alleviates Pressure Overload‐Induced Cardiac Fibrosis via Interfering TGF‐beta1 Signaling,” Journal of Molecular and Cellular Cardiology 116 (2018): 135–144. 29438665 10.1016/j.yjmcc.2018.02.006

[12] H. Port , C. M. Hausgaard , Y. He , et al., “A Novel Biomarker of MMP‐Cleaved Cartilage Intermediate Layer Protein‐1 Is Elevated in Patients With Rheumatoid Arthritis, Ankylosing Spondylitis and Osteoarthritis,” Scientific Reports 13, no. 1 (2023): 21717. 38066013 10.1038/s41598-023-48787-x PMC10709337

[13] W. Wang , J. Hao , S. Zheng , et al., “Association Between Cartilage Intermediate Layer Protein and Degeneration of Intervertebral Disc: A Meta‐Analysis,” Spine (Phila Pa 1976) 41, no. 20 (2016): E1244–E1248. 27359356 10.1097/BRS.0000000000001749

[14] S. Seki , N. Tsumaki , H. Motomura , et al., “Cartilage Intermediate Layer Protein Promotes Lumbar Disc Degeneration,” Biochemical and Biophysical Research Communications 446, no. 4 (2014): 876–881. 24631904 10.1016/j.bbrc.2014.03.025

[15] F. Lv , V. Y. Leung , S. Huang , Y. Huang , Y. Sun , and K. M. Cheung , “In Search of Nucleus Pulposus‐Specific Molecular Markers,” Rheumatology (Oxford) 53, no. 4 (2014): 600–610. 24049099 10.1093/rheumatology/ket303

[16] Q. Xiang , J. Wang , Z. Cheng , et al., “Hsa_circ_0001946 Ameliorates Mechanical Stress‐Induced Intervertebral Disk Degeneration via Targeting miR‐432‐5p and SOX9,” Spine (Phila Pa 1976) 48, no. 23 (2023): E401–E408. 37555796 10.1097/BRS.0000000000004777 PMC10624407

[17] Y. Teng , Y. Huang , H. Yu , et al., “Nimbolide Targeting SIRT1 Mitigates Intervertebral Disc Degeneration by Reprogramming Cholesterol Metabolism and Inhibiting Inflammatory Signaling,” Acta Pharmaceutica Sinica B 13, no. 5 (2023): 2269–2280. 37250166 10.1016/j.apsb.2023.02.018 PMC10213799

[18] C. L. Zhang , Q. Zhao , H. Liang , et al., “Cartilage Intermediate Layer Protein‐1 Alleviates Pressure Overload‐Induced Cardiac Fibrosis via Interfering TGF‐β1 Signaling,” Journal of Molecular and Cellular Cardiology 116 (2018): 135–144. 29438665 10.1016/j.yjmcc.2018.02.006

[19] K. Johnson , D. Farley , S. I. Hu , and R. Terkeltaub , “One of Two Chondrocyte‐Expressed Isoforms of Cartilage Intermediate‐Layer Protein Functions as an Insulin‐Like Growth Factor 1 Antagonist,” Arthritis and Rheumatism 48, no. 5 (2003): 1302–1314. 12746903 10.1002/art.10927

[20] Z. M. Liu , C. C. Lu , P. C. Shen , et al., “Suramin Attenuates Intervertebral Disc Degeneration by Inhibiting NF‐κB Signalling Pathway,” Bone & Joint Research 10, no. 8 (2021): 498–513. 34372688 10.1302/2046-3758.108.BJR-2020-0041.R3 PMC8414441

[21] C. Tseng , B. Chen , Y. Han , et al., “Advanced Glycation End Products Promote Intervertebral Disc Degeneration by Transactivation of Matrix Metallopeptidase Genes,” Osteoarthritis and Cartilage 32, no. 2 (2024): 187–199. 37717904 10.1016/j.joca.2023.09.005

[22] G. Xie , C. Liang , H. Yu , and Q. Zhang , “Association Between Polymorphisms of Collagen Genes and Susceptibility to Intervertebral Disc Degeneration: A Meta‐Analysis,” Journal of Orthopaedic Surgery and Research 16, no. 1 (2021): 616. 34663366 10.1186/s13018-021-02724-8 PMC8522091

[23] H. W. Xu , X. Y. Fang , X. W. Liu , et al., “Alpha‐Ketoglutaric Acid Ameliorates Intervertebral Disk Degeneration by Blocking the IL‐6/JAK2/STAT3 Pathway,” American Journal of Physiology. Cell Physiology 325, no. 4 (2023): C1119–C1130. 37661920 10.1152/ajpcell.00280.2023

[24] D. D. Roberts and J. S. Isenberg , “CD47 and Thrombospondin‐1 Regulation of Mitochondria, Metabolism, and Diabetes,” American Journal of Physiology. Cell Physiology 321, no. 2 (2021): C201–C213. 34106789 10.1152/ajpcell.00175.2021 PMC8424672

[25] S. M. Julovi , B. Sanganeria , N. Minhas , K. Ghimire , B. Nankivell , and N. M. Rogers , “Blocking Thrombospondin‐1 Signaling via CD47 Mitigates Renal Interstitial Fibrosis,” Laboratory Investigation 100, no. 9 (2020): 1184–1196. 32366943 10.1038/s41374-020-0434-3

[26] T. Gwag , E. Ma , C. Zhou , and S. Wang , “Anti‐CD47 Antibody Treatment Attenuates Liver Inflammation and Fibrosis in Experimental Non‐Alcoholic Steatohepatitis Models,” Liver International 42, no. 4 (2022): 829–841. 35129307 10.1111/liv.15182 PMC9101015

[27] P. Wang , C. Yang , J. Lu , Y. Ren , D. Goltzman , and D. Miao , “Sirt1 Protects Against Intervertebral Disc Degeneration Induced by 1,25‐Dihydroxyvitamin D Insufficiency in Mice by Inhibiting the NF‐kappaB Inflammatory Pathway,” Journal of Orthopaedic Translation 40 (2023): 13–26. 37200907 10.1016/j.jot.2023.04.003 PMC10185703

[28] Y. Zhao and A. Li , “miR‐19b‐3p Relieves Intervertebral Disc Degeneration Through Modulating PTEN/PI3K/Akt/mTOR Signaling Pathway,” Aging (Albany NY) 13, no. 18 (2021): 22459–22473. 34554926 10.18632/aging.203553 PMC8507280

[29] B. Lu , X. Chen , H. Chen , et al., “Demethoxycurcumin Mitigates Inflammatory Responses in Lumbar Disc Herniation via MAPK and NF‐kappaB Pathways In Vivo and In Vitro,” International Immunopharmacology 108 (2022): 108914. 35729841 10.1016/j.intimp.2022.108914

