# Supplementary material for: Cartilage Intermediate Layer Protein‐1 Promotes Extracellular Matrix Degeneration via Interacting With CD47
Source: J Cell Mol Med. 2025 Mar 23;29(6):e70506. doi: 10.1111/jcmm.70506 (PMC11930641; doi:10.1111/jcmm.70506)
Supplement: Supplementary file 1 — Figure S1. [file JCMM-29-e70506-s001.docx]

Supplementary figures


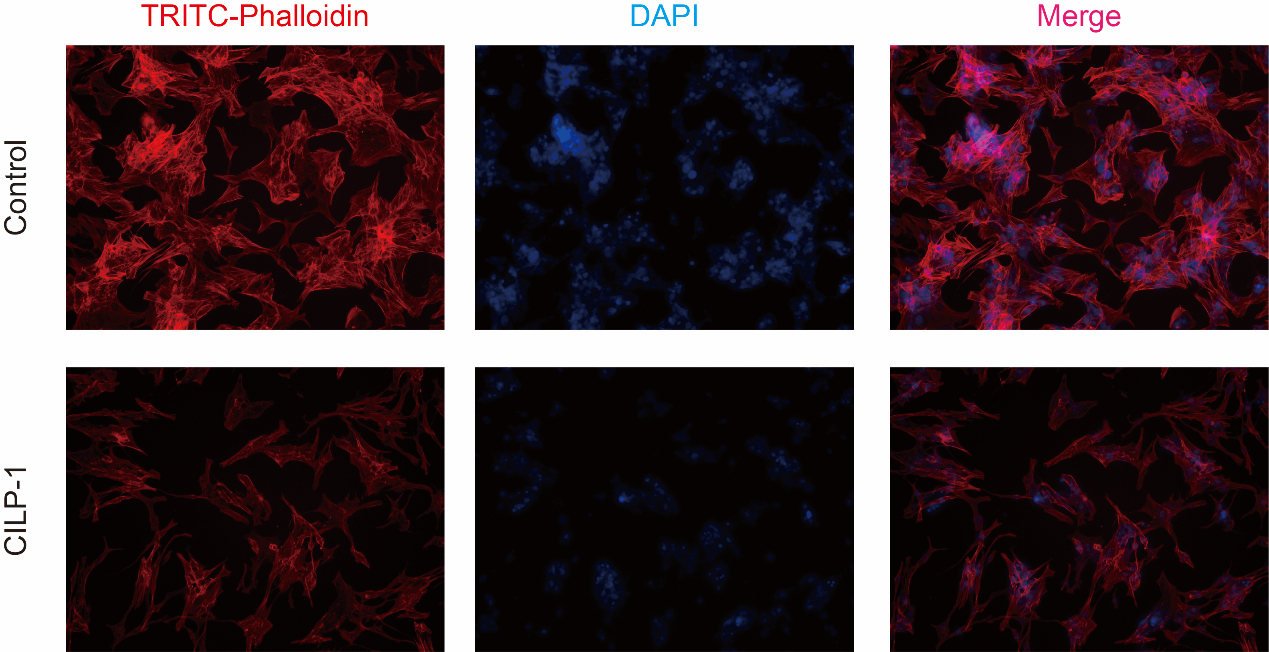


Figure S1. the TRITC-Phalloidin staining for F-actin of NP cells with and without treatment of CILP-1.


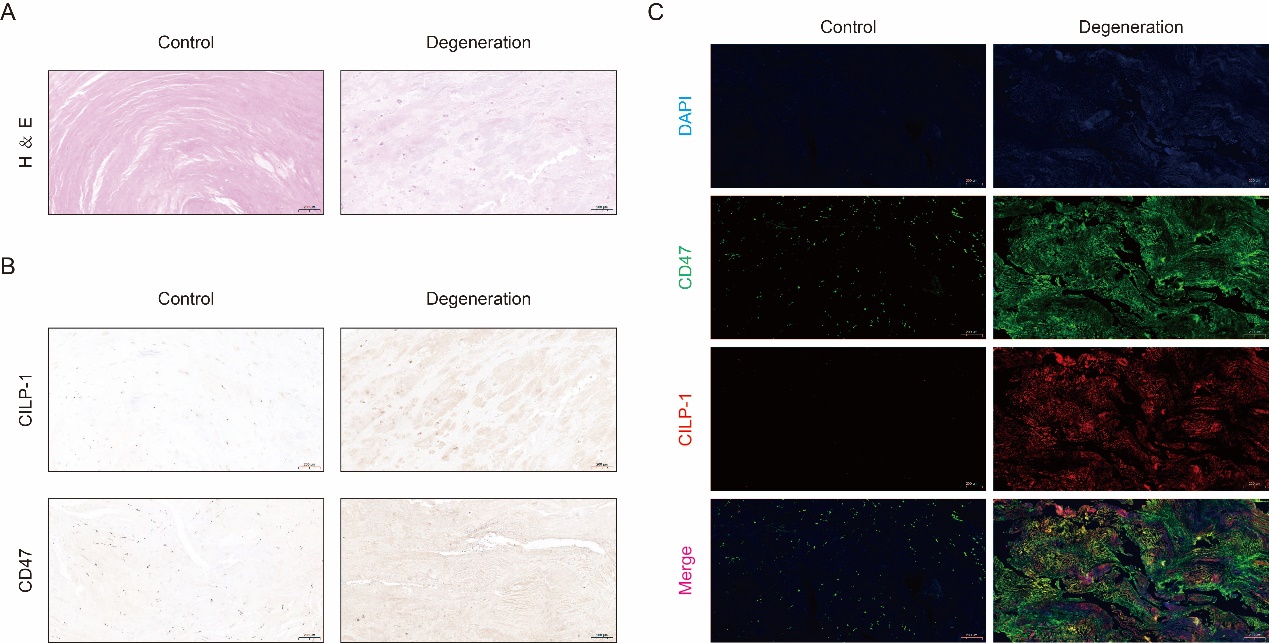


Figure S2. A: the H&E staining of healthy disc tissue and degenerative disc tissue; B: the immunohistochemistry staining for CILP-1 and CD47 of healthy disc tissue and degenerative disc tissue; C: the immunofluorescence staining for CILP-1 and CD47 of healthy disc tissue and degenerative disc tissue.


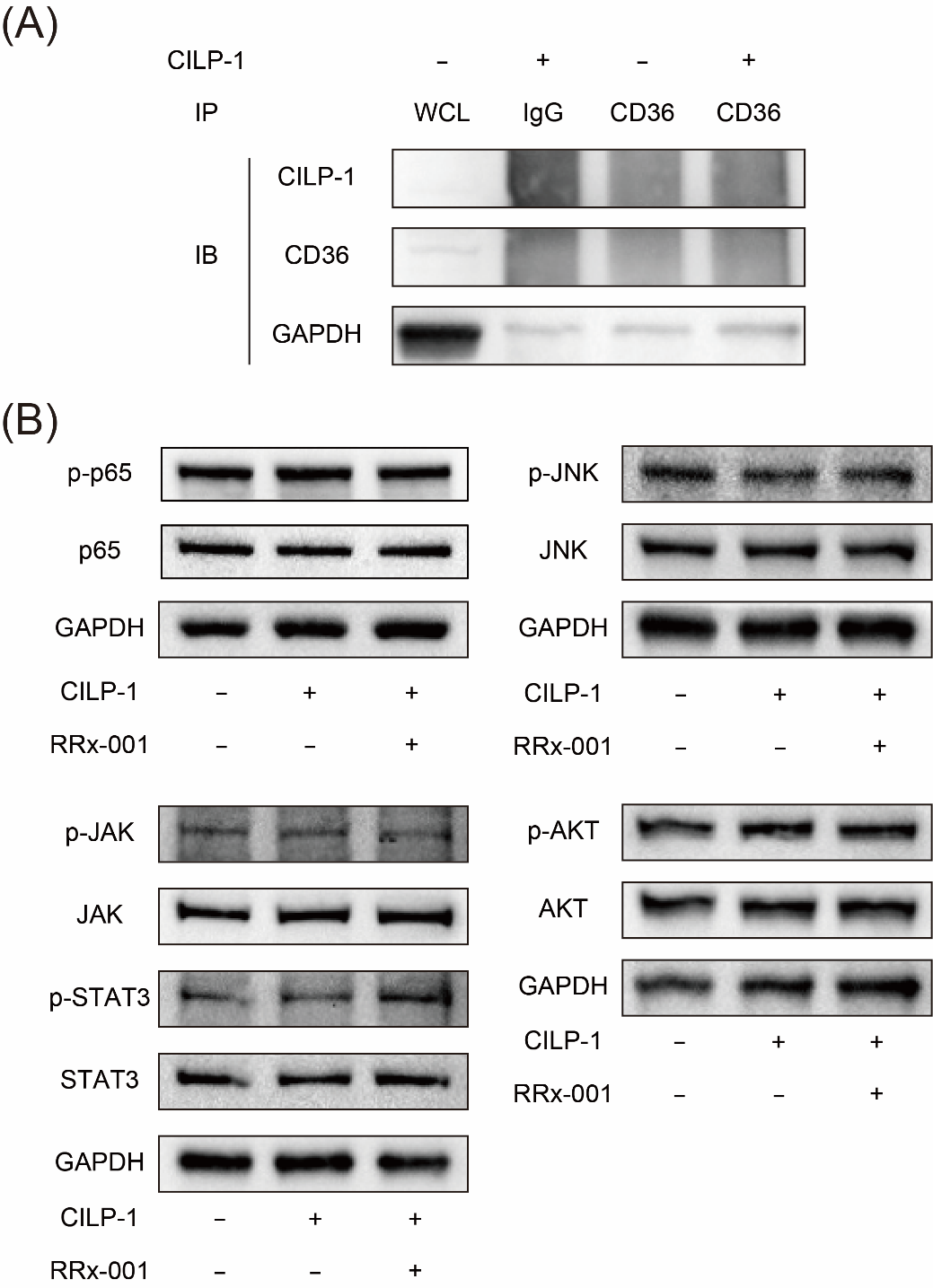


Figure S3. A: Co-IP result of CILP-1 and CD36; B: Western bolt results of signaling pathways in NP cells after treatment with CILP-1 and CD47 inhibitor.
