# Supplementary material for: Cartilage Intermediate Layer Protein‐1 Promotes Extracellular Matrix Degeneration via Interacting With CD47
Source: J Cell Mol Med. 2025 Mar 23;29(6):e70506. doi: 10.1111/jcmm.70506 (PMC11930641; doi:10.1111/jcmm.70506)
Supplement: Supplementary file 2 — Figure S4. [file JCMM-29-e70506-s003.docx]

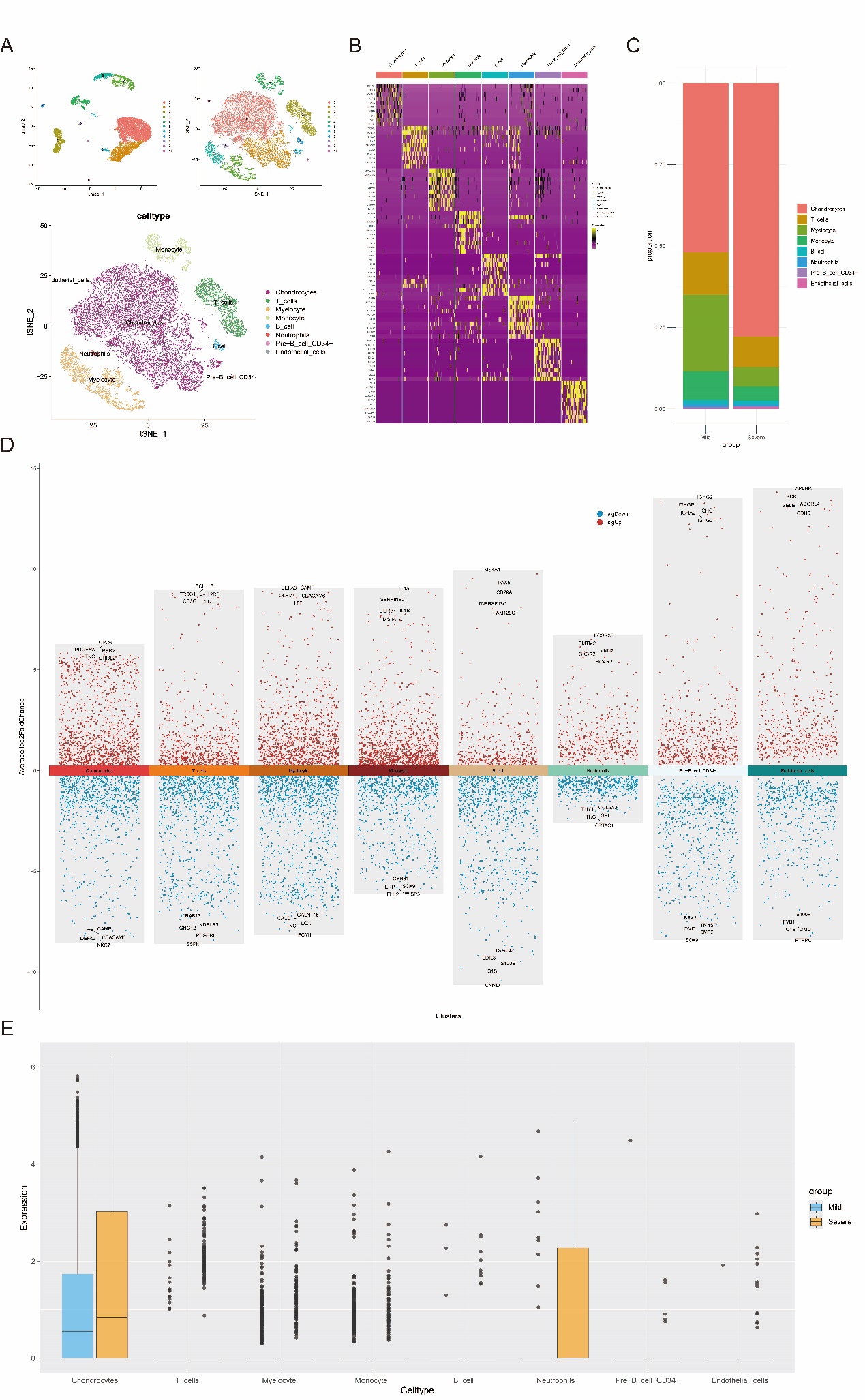


Figure S4. Results of single-cell data analysis

A: the results of UMAP and TSNE clustering; B: heatmap of differential genes; C: column chart of cell proportion distribution; D: volcano map of differential genes; E: expression of CILP showed by the boxplot.
