# Supplementary material for: Cartilage Intermediate Layer Protein‐1 Promotes Extracellular Matrix Degeneration via Interacting With CD47
Source: J Cell Mol Med. 2025 Mar 23;29(6):e70506. doi: 10.1111/jcmm.70506 (PMC11930641; doi:10.1111/jcmm.70506)
Supplement: Supplementary file 3 — Table S1. [file JCMM-29-e70506-s002.docx]

|  | Forward | Reverse |
| --- | --- | --- |
| Aggrecan | CTACCAGTGGATCGGCCTGAA | CGTGCCAGATCATCACCACA |
| MMP3 | ATTCCATGGAGCCAGGCTTTC | CATTTGGGTCAAACTCCAACTGT |
| ADAMTS5 | GGACCTACCACGAAAGCAGATC | GCCGGGACACACGGAGTAC |
| ADAMTS4 | ACTGGTGGTGGCAGATGACA | TCACTGTTAGCAGGTAGCGCTTT |
| MMP1 | CCAGGTATTGGAGGGGATGC | GTCCAAGAGAATGGCCGAGT |
| COL3A1 | AATTTGGTGTGGACGTTGGC | TTGTCGGTCACTTGCACTGG |
| COL1A1 | TACAGCGTCACTGTCGATGGC | TCAATCACTGTCTTGCCCCAG |
| COL2A1 | CCAGATGACCTTCCTACGCC | TTCAGGGCAGTGTACGTGAAC |
| RUNX2 | TGGTTACTGTCATGGCGGGTA | TCTCAGATCGTTGAACCTTGCTA |
| SOX9 | AGCGAACGCACATCAAGAC | CTGTAGGCGATCTGTTGGGG |
| MMP13 | AGACTTCCCAGGAATTGGTGA | ACGGTTACTCCAGATGCTGT |
| IL6 | ACTCACCTCTTCAGAACGAATTG | CCATCTTTGGAAGGTTCAGGTTG |
| Sirt1 | CCATACCCCATGAAGTGC | GCAGATGAGGCAAAGGTT |
| PTEN | GAGGGCCAGGTCATAAATAA | ACCATAAAATGTAAGCAAGGC |
| p27 | CTATCTGCTGCGCGGTT | CGAGTTCCTGACAAGCCA |
| CCL3 | TGCCAAACAGCCACACT | GGGGACAGGGGAACTCT |
| CCL4 | CGCATCTCCTCCATACTCA | GGGACACTTATCCTTTGGC |
| CA12 | TGGCATTCTTGGCATCTGTA | TTGGTGGCTGGCTTGTAAAT |
| CDH2 | TCAGGCGTCTGTAGAGGCTT | ATGCACATCCTTCGATAAGACTG |
| KRT19 | GCGAGCTAGAGGTGAAGATC | CGGAAGTCATCTGCAGCCA |
| IBSP | AACAAGGCATAAACGGCACCAGTA | CGGTAATTGTCCCCACGAGGTT |

Table S1 Primers Used in This Study.
